# The impact of natural disasters on healthcare and surgical services in low- and middle-income countries

**DOI:** 10.1097/MS9.0000000000001041

**Published:** 2023-07-06

**Authors:** Abdus Salam, Andrew A. Wireko, Riaz Jiffry, Jyi C. Ng, Heli Patel, Muhammad J. Zahid, Aashna Mehta, Helen Huang, Toufik Abdul-Rahman, Arda Isik

**Affiliations:** a Department of Surgery, Khyber Teaching Hospital; b Department of Surgery, Hayatabad Medical Complex, Peshawar, Pakistan; c Royal College of Surgeons in Ireland, University of Medicine and Health Sciences, Dublin, Ireland; d Faculty of Medicine and Health Sciences, University of Putra Malaysia, Serdang, Malaysia; e Nova Southeastern University Dr. Kiran C. Patel College of Allopathic Medicine, Florida, USA; f Sumy State University, Sumy, Ukraine; g Faculty of Medicine, University of Debrecen, Debrecen, Hungary; h Department of General Surgery, Istanbul Medeniyet University, Istanbul, Turkey

## Introduction

Humanitarian settings, such as natural disasters, cause human suffering and destruction, especially in low- and middle-income countries (LMICs). In a challenging scenario, such as the aftermath of a natural disaster, health systems can collapse under the burden of casualties. However, qualified surgical care remains crucial in LMICs when exposed to the debilitating consequences of natural disasters. With LMICs experiencing three times more fatalities in the event of natural disasters compared to high-income countries, it is essential to establish minimum standards of care when initiating surgical care activities^1^.

Médecins Sans Frontières (MSF) and Foreign Field Hospitals (FFHs) exemplify the implementation of the ‘do no harm’ principle, especially in a challenging situation to ensure the best possible quality surgical care to the infected population^2,3^. While natural disasters present unique scientific challenges and conditions, a report by FFH reports a lack of detailed information on the activities pursued by the healthcare teams^3^. The inaccessibility and time sensitivity of surgical care further exacerbate the effects of natural disasters, and thus it becomes crucial to enforce protocols for standard operating procedures to provide guidance to healthcare professionals.

This editorial sheds light on climate change issues, natural disasters, and their effects on healthcare delivery, particularly surgical care in LMICs, as well as potential recommendations.

## Current climate situation and natural disasters in LMICs

From an annual total of 33 catastrophes in 1960 to a peak of 441 disasters in 2000, there have been more than 11 000 natural disasters documented since 1960. As evidenced by the 510 837 deaths and 3.9 billion people affected by 6681 natural disasters between 2000 and 2019, the rising death rate demonstrates how populations remain vulnerable to natural disasters, particularly in LMICs. According to the analysis of the Emergency Events Database (EM-DAT), low-income nations experience more than three times as many fatalities from disasters as high-income countries do. Though lower-income nations had 44% of the disasters, they suffered 68% of the fatalities, compared to higher-income countries’ 56% of disasters and 32% of fatalities^1^.

While floods have occurred five times more frequently than in the 1980s, with over 3000 major incidents, climate disasters are responsible for about 90% of the 7345 disasters documented in EM-DAT since 2000^4^. Climate hazards cause significant morbidity and mortality in vulnerable countries because LMICs lack adequate preparedness and disaster management strategies. Between 1971 and 2016, Nepal experienced over 26 000 natural disasters, resulting in over 43 000 deaths and 83 000 injuries^5^. A total of 432 major disasters were reported in 2021, which is significantly more than the average of 357 disastrous events per year between 2001 and 2020. With 223 incidents, floods predominated these catastrophes, exceeding the 163 annual flood events averaged during the period from 2001 to 2020^6^.

The disaster database (EM-DAT) documented 187 catastrophes from natural disasters in 79 different countries during the first half of 2022, with their effects amounting to more than 6000 deaths, more than 50 million people impacted, and total damage estimates above 40 billion dollars. LMICs, among the most afflicted nations, saw the majority of the fatalities from natural catastrophes. This year, floods have affected millions of people in Pakistan, Bangladesh, and India. Over 33 million people have been affected by Pakistan’s most severe and widespread floods in 30 years, and roughly one-third of the nation is currently underwater. Outside of Asia, devastating floods have also struck South Africa, Brazil, Niger, Guatemala, and Bolivia. Other significant catastrophes include the earthquake in Afghanistan, which claimed more than 1000 lives; tropical storm Megi in the Philippines, which claimed 289 deaths; and Cyclone Batsirai in Madagascar, which is estimated to have killed 121 people. Storms are the second most common and destructive calamity after floods^7^.

## Impact of natural disasters on healthcare and surgical care in LMICs

Natural disasters pose significant challenges to the provision and accessibility of healthcare services, especially in low-income countries. Events like floods, earthquakes, and hurricanes can inflict damage on critical infrastructure, including healthcare facilities, making it difficult to provide care to those in need. The aftermath of these disasters can lead to power outages, water shortages, road damage, and communication system interruptions, which can severely disrupt healthcare delivery. Consequently, healthcare providers face numerous obstacles that can significantly impact their ability to deliver timely and appropriate care to those affected.

One aspect of healthcare disruption during natural disasters that is often overlooked is the impact on healthcare workers. During these events, healthcare workers are often overworked and under immense stress, which can result in physical and emotional exhaustion. Such conditions can compromise their ability to deliver quality care, which can negatively impact patient outcomes. Moreover, healthcare workers may also be personally affected by the disaster, which can lead to disruptions in both their personal and professional lives.

Foreign medical teams (FMTs) are often deployed to assist with post-disaster response in LMICs. The decision to deploy FMTs is usually based on several factors, such as the failure of the local healthcare system, the need for temporary facilities, a lack of healthcare expertise at the local level, and the unavailability of local healthcare professionals. While the assistance provided by FMTs can be crucial, it is important to note that they typically arrive after the initial response to a disaster, which can occur hours to days after the primary incident system, the need for temporary facilities, a lack of healthcare expertise at the local level, and the unavailability of local healthcare professionals. While the assistance provided by FMTs can be crucial, it is important to note that they typically arrive after the initial response to a disaster, which can occur hours to days after the primary incident. Therefore, the local healthcare system’s reliance on FMTs can add to the vulnerability of healthcare provision in LMICs during natural disasters, and there is a need to ensure that local healthcare systems are strengthened to withstand the impact of natural disasters.

Case studies of recent natural disasters highlight the impact on healthcare and surgical care in LMICs. For instance, floods have destroyed health facilities in extreme cases, as demonstrated by the flooding in Tamale, Ghana, in July 2017, which caused the collapse of two major hospitals, the Tamale Teaching Hospital and the Central Hospital^8^. This inevitably increased the workload of other hospitals’ surgical and medical staff in Northern Ghana. Water entered the wards and operating rooms as a result of the flood, causing many health services, including surgical procedures, to be halted due to contamination concerns. Accra Hospital in Ghana reported significant losses of medical equipment, medicines, and other supplies stored in their warehouse during a similar flood in 2015^8^.

In 2015, Nepal was struck by a devastating earthquake that severely impacted the healthcare system. Giri *et al*.^9^ conducted a prospective observational study of patients who presented to Dhulikhel Hospital in Nepal following the 2015 earthquake. It took more than 3 days after the earthquake for international field hospitals to begin treating patients, so critical injury victims who required emergency surgical interventions had to rely solely on the surgical capacity of Dhulikhel Hospital. The authors reported that the surgeons at Dhulikhel Hospital performed 345 surgical procedures, almost all of which were orthopedic in nature. Although the local healthcare system could adequately treat most surgical emergencies, due to a lack of neurosurgical services at Dhulikhel Hospital, eight patients with internal head injuries had to be transferred to a hospital in Kathmandu. Furthermore, communication and transportation facilities were disrupted as a result of the earthquake, making it difficult to coordinate patient transfers to other health institutions^9^. As a result, Dhulikhel Hospital had to rely solely on its own healthcare workers and medical supplies to provide 24-hour surgical services.

Also, the earthquake that occurred in Haiti in January of 2010 left many nearby hospitals unsafe or destroyed and thus unavailable to provide emergency medical or surgical care^10^. Furthermore, it is critical to recognize the inherent baseline limitations in surgical care access in LMICs, which are only exacerbated during natural disasters. Bagguley *et al*.^11^ investigated the lengths of time patients waited for essential surgical care, known as Bellwether procedures, in a Timor Leste national referral hospital. The authors measured the endpoints using the Three Delays Framework and observed delays in all three stages of the framework for each Bellwether procedure.

Pakistan currently experienced severe flooding, which has caused the collapse of most roads and bridges in the Khyber Pakhtunkhwa province, rendering ~50% of its villages inaccessible^12^. As a result, the only way for medical and surgical teams to reach victims was to swim toward them. According to the WHO, nearly 900 healthcare institutions across Pakistan have been damaged, resulting in significant reductions in access to health services and shortages of critical medical supplies^12^. Pregnant women are among the many patient populations disproportionately affected by the floods; several health institutions in rural Sindh province are currently underwater, leaving pregnant women in these areas without facilities or medical professionals to assist them with parturition. Other challenges the floods have produced include increased rates of water and mosquito-borne illnesses such as cholera and malaria, as well as skin infections, particularly scabies and dermatomycoses^12^. Overall, natural disasters can have devastating effects on the healthcare system and surgical care provision in LMICs. It is crucial to understand the various impacts of these disasters, including their effects on healthcare workers, and to prepare and implement effective disaster response plans to mitigate the impact on vulnerable populations.

## Efforts

Having access to quick, high-quality surgical treatment is imperative, but not all countries offer the same level of surgical care. The WHO established structural and procedure standards for the delivery of surgical care in 2003, which was a significant step in the direction of standardized surgical care in LMICs district hospitals^13^.

After the catastrophic earthquake in Haiti in January 2010, which left many dead and injured and significant damage to the country’s healthcare infrastructure, the need for minimal requirements to deliver high-quality surgical care arose^2^. To prevent such catastrophes in the future, hospitals were constructed in rural areas of Haiti to provide access to emergency surgical care after natural disasters, solving the accessibility issue without overburdening the tertiary care hospitals while providing quality surgical care^10^.

Later in 2013, the WHO published guidelines on international minimum surgical care standards in the aftermath of a disaster, which include outpatient emergency care, inpatient surgical emergency care, and inpatient referral care, based on their ability to deliver surgical care^14^.

## Recommendations

### Need for baseline research in LMICs

It is difficult to estimate the surgical need associated with natural disasters in LMICs due to a lack of research on surgically manageable conditions in disaster settings. As the frequency and severity of major disasters increase, particularly in LMICs, millions of people will require surgical care in addition to their basic needs. The need for surgical care for those affected by climate-related natural disasters is expected to rise in countries with limited surgical capacity. Estimates of surgical needs are thus critical because they facilitate surgical advocacy while also guiding national policy and humanitarian relief programs and involving stakeholders. These estimates are especially important for LMICs, which are the least prepared to address surgical care needs due to significant human and material resource shortages. To achieve this, we recommend that health ministries and healthcare facilities in LMICs establish a platform to identify shortcomings, evaluate, and track their performance for quality improvement. These platforms should involve various stakeholders, including healthcare workers, policy-makers, and disaster management organizations. The metrics for quality assessment should include the availability of essential surgical equipment, supplies, and trained personnel, as well as the capacity to provide emergency surgical care during natural disasters. The allocation of resources toward mitigation strategies should be based on the identified shortcomings and available resources, and it should involve a multi-stakeholder approach that includes government funding, international aid, and community participation.

### Improving disaster preparedness in LMICs

To improve disaster preparedness at the local and national levels, multidisciplinary efforts are required. Healthcare workers in disaster-prone areas should be prepared to handle emergency situations. Annual emergency preparedness simulations and modules should be conducted to provide medical personnel with the skills and knowledge needed to respond to disasters in a coordinated, timely, safe, and effective manner. These simulations should also involve community participation and include the development of disaster response plans that take into account the unique needs of the community and the healthcare facilities.

### Building disaster-resilient surgical care systems in LMICs

Future disaster victims are likely to suffer from unfavorable outcomes unless significant measures are taken to enhance national capacity and global humanitarian surgical preparedness in vulnerable countries. To build a disaster-resilient surgical care system in LMICs, we suggest a multi-faceted approach that addresses both the built environment and the availability of resources. Resilient in terms of the built environment refers to the ability of healthcare facilities to endure natural disasters and continue providing surgical care during and after such events. This involves ensuring that the facilities are designed and constructed to withstand anticipated natural hazards in the area, such as earthquakes, floods, and hurricanes. It also involves having backup power and water supplies, as well as emergency communication systems. Resilient in terms of resources refers to the availability of trained surgical personnel, equipment, and supplies to provide emergency surgical care during and after natural disasters. This involves training and equipping healthcare workers with the skills and knowledge needed to respond to disasters, as well as ensuring that there is an adequate supply of essential surgical equipment and supplies.

To establish a disaster-resilient surgical care system in LMICs, the WHO health systems framework can be employed as a guide. The framework consists of six building blocks: service delivery, health workforce, information system, access to necessary medicine, financing, and leadership and governance. Health ministries and healthcare facilities in LMICs can use this framework to identify and address inadequacies in their surgical care systems and develop strategies to enhance their disaster preparedness. It is essential to recognize that building disaster-resilient surgical care systems necessitates a multi-stakeholder approach that involves government funding, international aid, and community participation.

## Conclusion

Natural disasters have become more common as a result of global climate change. Although it is impossible to prevent natural disasters, it is possible to reduce the devastating impact of natural disasters on healthcare services. LMICs are especially vulnerable to the consequences of natural disasters, so global efforts to improve outcomes are critical.

## Ethical approval

Not applicable.

## Consent

Not applicable.

## Source of funding

The authors did not receive any financial support for this work.

## Author contribution

A.S.: conceptualized the ideas. All authors were involved in the writing of the initial draft. A.S., A.A.W., R.J., J.C.N., H.P., M.J.Z., A.M., H.H., T.A.-R., and A.I.: reviewed and edited the manuscript.

## Conflicts of interest disclosure

The authors declare that there are no conflicts of interest.

## Research registration unique identifying number (UIN)

Not applicable.

## Guarantor

Andrew Awuah Wireko.

## Data availability statement

No data available.

## Provenance and peer review

Not commissioned, externally peer-reviewed.

## Acknowledgements

None.
